# Spatio-temporal analysis of the incidence of colorectal cancer in Guangzhou, 2010–2014

**DOI:** 10.1186/s40880-017-0231-6

**Published:** 2017-07-28

**Authors:** Ke Li, Guo-Zhen Lin, Yan Li, Hang Dong, Huan Xu, Shao-Fang Song, Ying-Ru Liang, Hua-Zhang Liu

**Affiliations:** Department of Biostatistics and Cancer Registration, Guangzhou Center for Disease Control and Prevention, Guangzhou, 510440 Guangdong P. R. China

**Keywords:** Colorectal cancer, Spatial analysis, Spatial autocorrelation, Spatio-temporal clustering

## Abstract

**Introduction:**

Colorectal cancer (CRC) is a common type of neoplasm. This study examined the spatio-temporal distribution of the CRC incidence in Guangzhou during 2010–2014.

**Methods:**

Colorectal cancer incidence data were obtained from the Guangzhou Cancer Registry System. Spatial autocorrelation analysis and a retrospective spatio-temporal scan were used to assess the spatio-temporal cluster distribution of CRC cases.

**Results:**

A total of 14,618 CRC cases were registered in Guangzhou during 2010–2014, with a crude incidence of 35.56/100,000 and an age-standardized rate of incidence by the world standard population (ASRIW) of 23.58/100,000. The crude incidence increased by 19.70% from 2010 (32.88/100,000) to 2014 (39.36/100,000) with an average annual percentage change (AAPC) of 4.33%. The AAPC of ASRIW was not statistically significant. The spatial autocorrelation analysis revealed a CRC incidence hot spot in central urban areas in Guangzhou City, which included 25 streets in southwestern Baiyun District, northwestern Haizhu District, and the border region between Liwan and Yuexiu Districts. Three high- and five low-incidence clusters were identified according to spatio-temporal scan of CRC incidence clusters. The high-incidence clusters were located in central urban areas including the border regions between Baiyun, Haizhu, Liwan, and Yuexiu Districts.

**Conclusions:**

This study revealed the spatio-temporal cluster pattern of the incidence of CRC in Guangzhou. This information can inform allocation of health resources for CRC screening.

## Background

Globally, colorectal cancer (CRC) ranked third in cancer incidence and fourth in cancer deaths in 2013, when an estimate of 1,600,000 new cases and 771,000 deaths occurred [1]. CRC resulted in the loss of 15,800,000 disability-adjusted life years in 2013, 56% of which were lost in developing countries and 44% in developed countries [1]. The incidence of CRC was high in Australia/New Zealand, Europe, and North America, but low in Africa and South Central Asia [2]. The incidence was higher in men than in women in most parts of the world [1, 2]. It is increasing in certain countries where the risk has been historically low, most notably in West Asia (Kuwait and Israel) and East Europe (Czech Republic and Slovakia) [2]. Trends in high-risk/high-income countries have varied over the past 20 years. The decrease in CRC incidence in the United States is confined to those aged 50 years and older, which primarily reflects the increase in screening and removal of precancerous adenomas [2]. In China, it is predicted that there will be about 376,000 newly diagnosed CRC cases and 190,000 deaths in 2015 [3]. The age-standardized rate (ASR) of incidence in China increased from 2000 to 2011 [3].

No single risk factor is recognized as accounting for most cases of CRC. Apart from age and male sex, the following risk factors (which often co-occur and interact) have been identified and established in epidemiological studies: family history of CRC, smoking, excessive alcohol intake, consumption of red and processed meat, and diabetes [4]. However, the risk factors for CRC might be different in Guangzhou, China.

Colorectal cancer screening was implemented in Guangzhou since 2015. This study aimed to explore the spatio-temporal distribution pattern of CRC using the information obtained from the Guangzhou Cancer Registry System. The findings will be useful in exploring the risk factors associated with the distribution of CRC, and will both provide etiologic information and facilitate decision making for effective implementation of CRC screening.

## Methods

### Data source

Colorectal cancer incidence data were obtained from the Guangzhou Cancer Registry, which was established in 1998 and covers all permanent residents of Guangzhou City. In this registry, all cancer cases diagnosed in 120 qualified hospitals (those with tumor diagnosis and treatment qualifications) were requested to be reported through a network direct reporting system. For each incident cancer case, information including registered identification number (ID), medical ID, China Identity Card Number (unique for each resident), name, sex, birth date, occupation, ethnicity, resident permanent address, phone number, cancer site, ICD-10 code, basis for diagnosis, treatment, prognosis and pathologic report if available, ICD-O-3 code, hospital, and the diagnosing physician’s name were registered. All cases were distributed to Community Health Service Centers for follow-up since 2010. The physicians in the Community Health Service Centers checked and supplemented the data (particularly the resident’s address). All cancers of the colon and rectum (ICD-10 codes C18–20) were included in the analysis. Cases with no or an incorrect address were excluded from the spatial and spatial–temporal analyses.

Population data were from the 2010 National Population Census [5], which covers the population of every street in Guangzhou, and from the 2010–2014 Guangzhou Bureau of Public Health and Statistics report [6], which covers the population of every district in Guangzhou. The population of each street during 2011–2014 was calculated as the product of the district population in 2011–2010 and the proportion of people residing on each street in 2010.

A map of the 167-street (town) administrative division in 2010 was obtained from the National Geographic Center of China. The 1984 World Geodetic System (WGS) map projection coordinating system from Defense Mapping Agency (DMA) was used. Although several streets were merged or divided between districts during 2010–2014, the 2010 street division was still used. The data were integrated in WGS, and ArcGis 10.2 software (Esri, Redlands, CA, USA) was used for mapping and data visualization.

### Spatial cluster analysis

A spatial cluster analysis of CRC cases was performed using spatial autocorrelation. A spatial cluster model of Guangzhou was established using the univariate Moran’s *I* tool in the OpenGeodata 1.2.0 software (Luc Anselin, Urbana, IL, USA) as follows [7]:

$$I = \frac{{n\mathop \sum \nolimits_{i} \mathop \sum \nolimits_{j} W_{ij} \left( {X_{i} - \overline{X} } \right)\left( {X_{j} - \overline{X} } \right)}}{{\mathop \sum \nolimits_{i} \mathop \sum \nolimits_{j} W_{ij} \mathop \sum \nolimits_{i} \left( {X_{i} - \overline{X} } \right)^{2} }}$$

where *X*_*i*_ = the crude incidence of cancer for the *i*th street; $\overline{X}$ = the mean crude incidence of cancer for all streets in the study area; *X*_*j*_ = the crude incidence of cancer for the *j*th street; *W*_*ij*_ = a weight parameter for the pair of streets *i* and *j* that represents proximity; and *n* = the number of streets.

Here, *I* > 0 indicates a clustered pattern (i.e., similar values are found together), *I* = 0 indicates a random pattern, and *I* < 0 indicates a dispersed pattern (i.e., high- and low-incidence values are scattered).

The cluster type and exact position were estimated using the univariate local Moran’s *I* tool (local indicators of spatial association, LISA) in the OpenGeodata 1.2.0 software. The local Moran’s I (*Ii*) for the *i*th city was calculated according to Anselin’s report [8] as follows:

$$I_{i} = \frac{{\left( {X_{i} - \overline{X} } \right)\mathop \sum \nolimits_{j} W_{ij} \left( {X_{j} - \overline{X} } \right)}}{{S^{2} }}$$

where *X*_*i*_ = the crude incidence of cancer for the *i*th street; $\overline{X}$ = the mean crude incidence of cancer for all streets in the study area; *X*_*j*_ = the crude incidence of cancer for the *j*th street; *W*_*ij*_ = a weight parameter for the pair of streets *i* and *j* that represents proximity; and *S* = the standard deviation of the crude incidence of cancer in the study area.

The local Moran’s I identifies statistically significant (at a 95% confidence level; *P* < 0.05) spatial clusters of streets with high or low crude cancer incidences. Clusters of streets with high crude cancer incidences (high–high) were considered “hot spots,” whereas clusters of streets with low crude cancer incidences (low–low) were considered “cold spots.” In addition, the local Moran’s I identifies streets with high crude cancer incidences that are surrounded mainly by streets with low crude cancer incidences (high-low), as well as streets with low crude cancer incidences that are surrounded mainly by streets with high crude cancer incidences (low–high).

### Spatio-temporal scan

The spatio-temporal cluster detection test for CRC incidence was retrospectively performed using spatial scan statistics. The scan parameters were as follows: the time range was from 2010 to 2014; the time interval was 1 year; the retrospective analysis metrics was space–time; the discrete scan statistics was Poisson; the maximum spatial cluster size was 10% of the population potentially at risk; the maximum temporal cluster size was 1 year; and the number of Monte Carlo simulations was restricted to 999. Then, the log-likelihood ratio (LLR) was calculated with the actual and theoretical incidences computed with the Poisson distribution in each scan window [9]. The LLR is proportional to

$$\left( {\frac{n}{E}} \right)^{n} \left( {\frac{N - n}{N - E}} \right)^{N - n} I$$

where *n* = the number of cancer cases within the scan window; *N* = the total number of cancer cases in the population; *E* = the expected number of cancer cases under the null hypothesis; and *I* = 1 when the scan window has a larger number of cancer cases than expected if the null hypothesis were true, and 0 otherwise.

The high-incidence cluster areas exhibited the higheset LLR values. Next, relative risk (RR) was calculated as the ratio of the incidence inside a cluster area to that outside the cluster area, and its significance was analyzed.

### Statistical analysis

The age-standardized rate of incidence by the world standard population (ASRIW) was estimated using the 1964 Segi’s world standard population. The descriptive analysis was carried out using Excel 2013 (Microsoft, Redmond, WA, USA). The spatial cluster analysis was performed by hypothesis testing of *Z* statistics for space aggregation indices using the OpenGeoda 1.2.0 software. The spatio-temporal scan analysis was conducted using the SaTScan 9.4.2 software developed by NCI. A trend test of the average annual percentage change (AAPC) was performed using the Joinpoint Regression Program, version 4.0.4, developed by National Cancer Institute (NCI, Boston, MA, USA). AAPC was calculated as follows:

$$\ln \left( {R_{y} } \right) = \alpha + \beta y,\,{\text{AAPC}} = \left( {e^{\beta } - 1} \right) \times 100,$$

where *Ry* is the incidence in year *y*.

## Results

### Basic information

During 2010–2014, a total of 14,618 CRC cases, including 8170 males and 6448 females, were registered in Guangzhou, accounting for approximately 14.21% of the total cancer cases. CRC ranked as the second most common type of cancer. The total crude incidence of CRC was 35.65/100,000, with an ASRIW of 23.58/100,000 (Table 1).

**Table 1:** Incidence of colorectal cancer in Guangzhou, China during 2010–2014

Year	Total (cases)	Crude incidence (/100,000)	ASRIW (/100,000)
2010	2624	32.88	23.20
2011	2788	34.30	23.43
2012	2874	35.05	23.25
2013	3026	36.46	23.43
2014	3306	39.36	24.47
Total	14,618	35.65	23.58
PC (%)		19.70	5.47
*P*		0.005	0.158

*ASRIW* age-standardized rate of incidence by the world standard population, *PC* percentage change

**Fig. 1:**
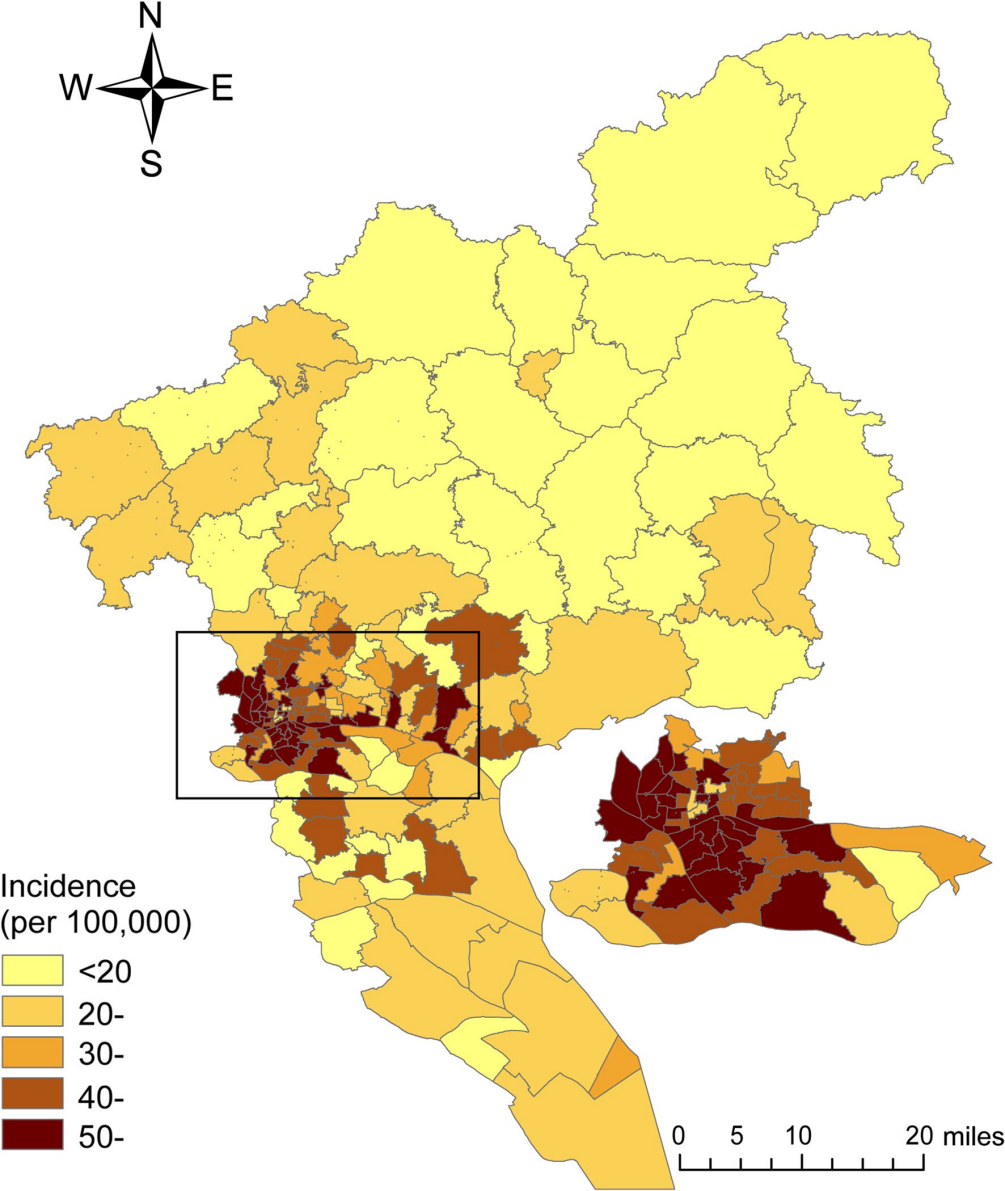
Geographic distribution of colorectal cancer incidence in Guangzhou, China during 2010–2014. The incidences of colorectal cancer in central urban areas are higher than those in surrounding suburban areas

The incidence of CRC was higher in urban than in rural areas, with the lowest on the Dalong Street (3.00/100,000) in Panyu District and the highest on the Beijing Street (81.95/100,000) in Yuexiu District (Fig. 1).

### Spatial autocorrelation

One hundred eighty cases were excluded from the spatial and spatial–temporal analyses due to the absence of an address or an incorrect address. The global spatial autocorrelation analysis of the cumulative incidence of CRC in Guangzhou during 2010–2014 showed a Moran’s I index of 0.527 (*Z* = 11.06, *P* < 0.001), suggesting that the spatial distribution of CRC in Guangzhou had spatial autocorrelation and cluster distribution patterns.

**Fig. 2:**
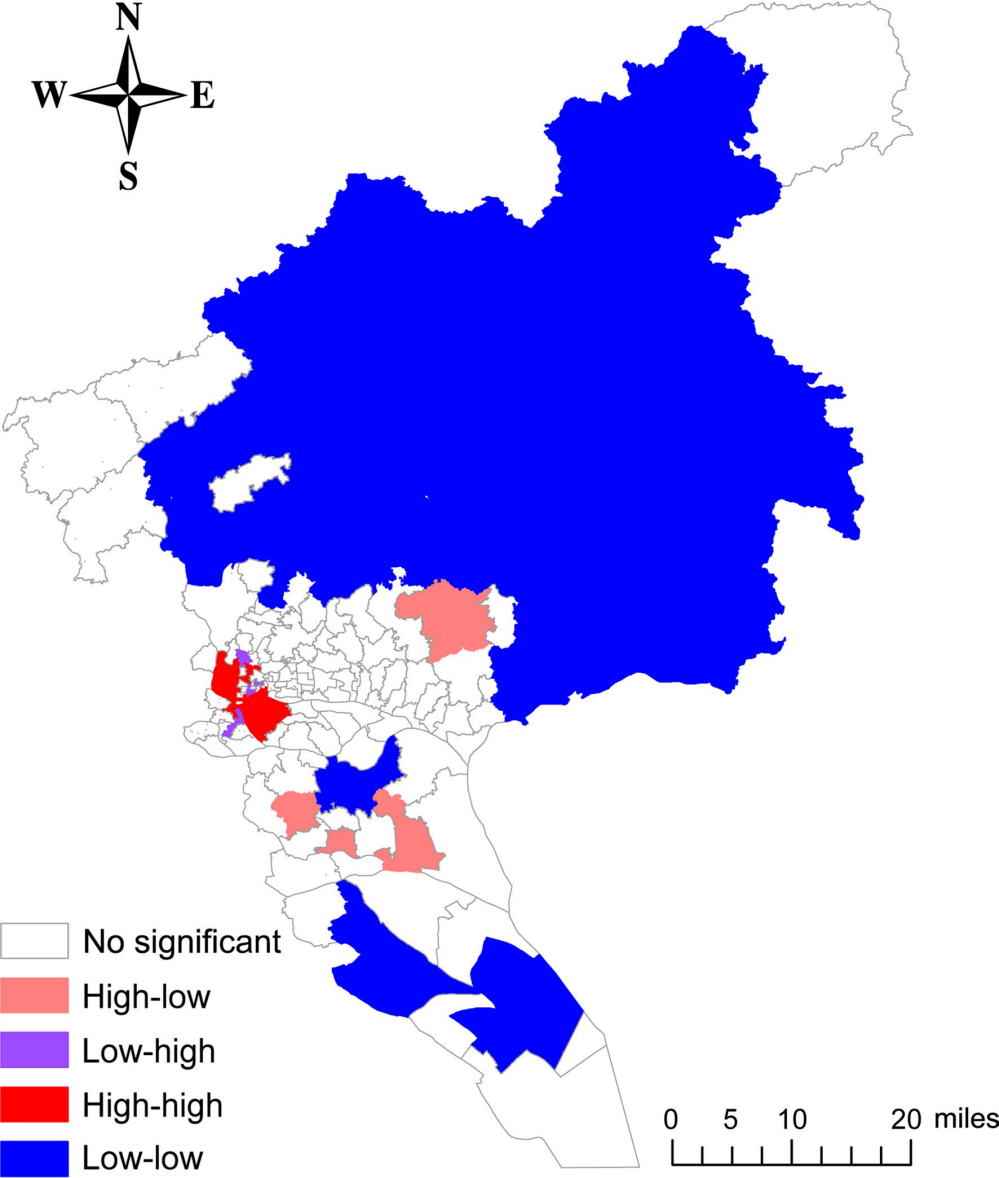
Local hot spot map for colorectal cancer incidence in Guangzhou, China during 2010–2014. High–high: clusters of streets with high incidences. Low-low: clusters of streets with low incidences. High–low: streets with high incidences surrounded mainly by streets with low incidences. Low–high: streets with low incidences surrounded mainly by streets with high incidences. High-high clusters include 25 streets in central urban areas. Low–low clusters include 30 streets in most suburban areas

The local autocorrelation analysis of the cumulative incidence of CRC in Guangzhou during 2010–2014 showed the presence of local hot spots and cold spots, with a local Moran’s I index of 0.527 (*Z* = 11.85, *P* < 0.001). Moreover, the LISA analysis demonstrated the presence of hot spots (high–high) of CRC incidence in central urban areas, which included 25 streets in southwestern Baiyun District, northwestern Haizhu District, and the border region of Liwan and Yuexiu Districts. Cold spots (low–low) were detected in most suburban areas, including 30 streets in Huadu District, Zengcheng and Conghua counties, and part of Panyu and Nansha Districts (Fig. 2).

### Temporal trend

The Joinpoint analysis indicated that the crude CRC incidence had a signifcant increasing trend, with an AAPC of 4.33% [95% confidence interval (CI) 2.60%–6.10%]. The AAPC of ASRIW (1.13%, 95% CI −0.50%–2.80%) was not statistically significant.

### Spatio-temporal clusters

**Fig. 3:**
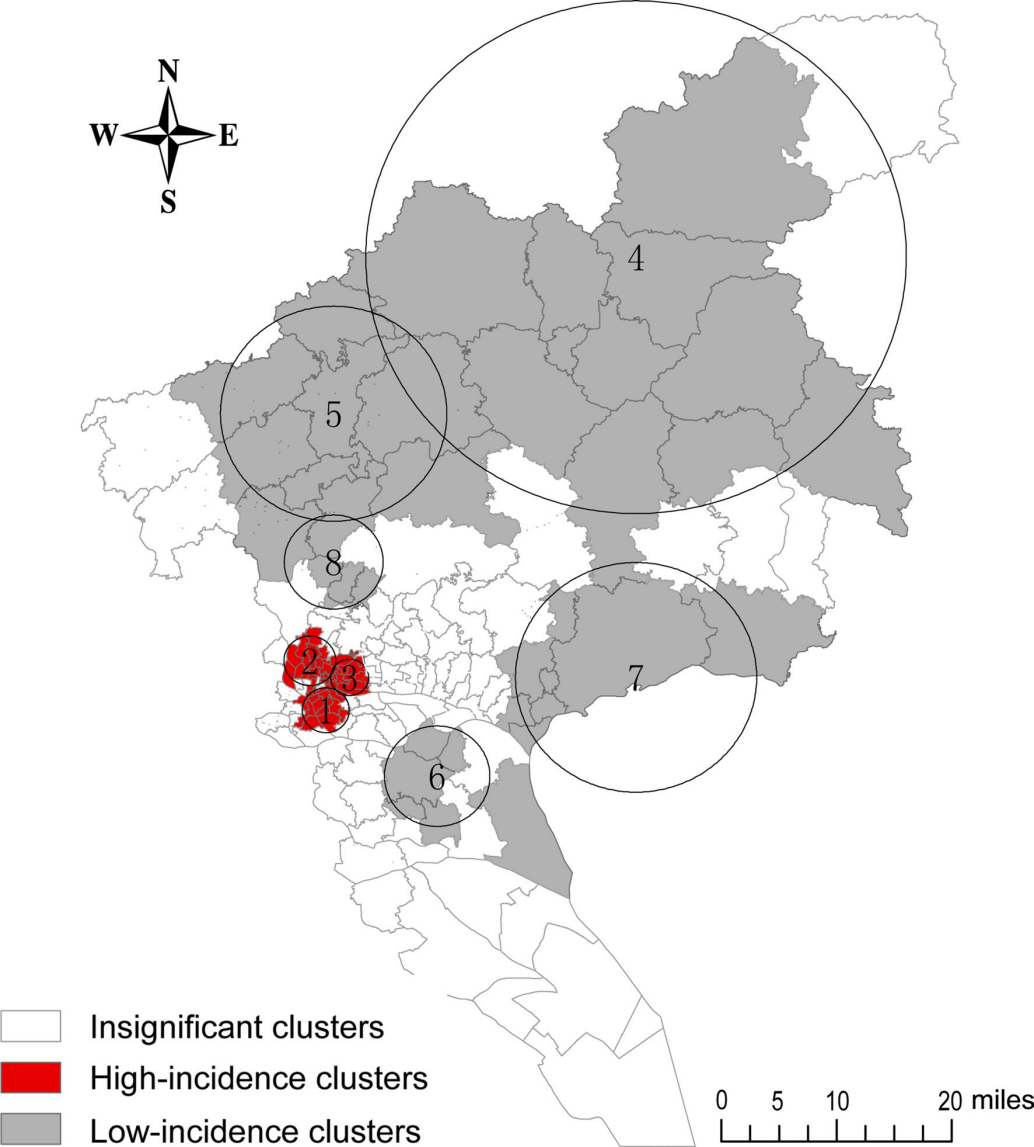
Spatio-temporal cluster map of colorectal cancer incidence in Guangzhou, China during 2010–2014. High 1–3 clusters: in the central urban areas, with significant higher incidences than those of other areas. Low 4–8 clusters: mainly in the surrounding suburban areas, with significant lower incidences than those of other areas. Insignificant clusters: the rest areas with dispersed incidences

The CRC incidence in Guangzhou during 2010–2014 was retrospectively scanned in a spatio-temporal manner by incorporating the dimensions of both time and space. The CRC incidences in three high- and five low-incidence clusters were significantly different from those in other clusters. High 1 cluster covered the border regions of Liwan, Yuexiu, and Haizhu Districts and included 16 streets (5 in Liwan, 1 in Yuexiu, and 10 in Haizhu Districts); this cluster had 570 cases in 2014 compared with 294 cases expected. High 2 cluster covered the border regions of Liwan, Yuexiu, and Baiyun Districts and included 20 streets (8 in Liwan, 8 in Yuexiu, and 4 in Baiyun Districts); in 2014, this cluster had 78.36% more cases than expected. High 3 cluster, a small area in western Yuexiu District, included 12 streets and had 54.38% more cases in 2014 than expected. Low 6 cluster, with the lowest incidence, covered western Panyu District and included 6 streets; it had 72.28% fewer cases in 2012 than expected. The other four low-incidence clusters were as follows: Low 4 cluster covered most of Conghua County and northern Zengcheng County (11 streets) in 2011; Low 5 cluster covered eastern Huadu District (8 streets) in 2010; Low 7 cluster covered southern Huangpu District, Luogang District, and Zengcheng County (8 streets) in 2011; Low 8 cluster covered northern Baiyun District (4 streets) in 2013. The distribution clusters are shown in Table 2 and Fig. 3.

**Table 2:** Colorectal cancer incidence statistics for the space–time analyses, Guangzhou, China during 2010–2014

Statistic	High-incidence cluster			Low-incidence cluster				
	1	2	3	4	5	6	7	8
Number of streets	16	20	12	11	8	6	8	4
Radius (km)	3.02	3.33	2.45	34.58	14.50	6.78	15.49	6.34
Observed cases	570	478	423	102	141	28	88	32
Expected cases	294	268	274	286	275	101	156	70
Year	2014	2014	2014	2011	2010	2012	2011	2013
RR	1.98	1.81	1.56	0.35	0.51	0.28	0.56	0.46
LLR	104.3	67.8	35.8	80.2	40.4	37.3	17.9	12.8
*P* value	<0.001	<0.001	<0.001	<0.001	<0.001	<0.001	<0.001	0.006

*RR* relative risk, *LLR* log likelihood ratio

## Discussion

In the present study, we found that CRC was the second most common type of cancer in Guangzhou, with an ASRIW of 23.58/100,000, which is lower than the average in more-developed and higher than that in less-developed regions in China [10]. We also found that the crude incidence of CRC increased significantly from 2010 to 2014, whereas the ASRIW stabilized. These findings are consistent with those during 2000–2011 [11]. A variety of factors can affect temporal trends, of which age may be the most important. Changes in lifestyle, such as a western diet lifestyle and less physical exercise, might also be related to the observed increasing trend.

Spatial analysis of geocoded cases of disease is useful in epidemiology. One health-focused application of spatial analysis is cluster detection. Using cluster detection to identify geographic areas with high-risk populations and then screening those populations for disease can improve cancer control [12]. Spatial scan statistics and LISA are widely used spatial analysis methods for detecting clusters of patients with a specific disease. Several previous studies have compared these methods in terms of identifying spatial clusters of patients with a specific disease and suggested these methods to be complementary [13–17]. Our analysis using both methods showed a nonrandom distribution of CRC incidence in Guangzhou. CRC incidence was higher in the central urban areas (the border regions between Baiyun, Haizhu, Liwan, and Yuexiu Districts). In contrast, the CRC incidences in suburban areas (Huadu District, Zengcheng and Conghua Counties, and parts of Panyu and Nansha Districts) was lower than those in other areas.

The causes of this spatial distribution of CRC incidence in Guangzhou are unclear, but they may be due to differences in risk factors such as a low-fiber, high-fat diet, increasing rate of obesity, fast-food consumption, mechanized lifestyles, and low levels of physical activity. Most central urban areas are wealthier than suburban areas. The incidence of CRC is high among populations with high socioeconomic status (SES) in Europe, Australia, and South Korea but low among populations with high SES in the US and Canada [18]. In developing countries, patients with high SES have a high incidence of CRC [19–21]. In the US, high SES was associated with high CRC incidence and CRC-related mortality during 1973–1997, but the opposite trend was noted since 1998 [22]. Several mechanisms may be involved in the link between CRC and SES. First, lifestyle factors such as physical activity and diet may be related to SES. Second, the CRC screening rate varies markedly among countries; in the US and Canada, screening rates were higher in high-SES populations than in low-SES populations [18]. Guangzhou, the capital of Guangdong Province, China, has undergone rapid economic development and continuous improvement in living standards. A higher proportion of the population in high-SES areas tends to adopt a western lifestyle than that in low-SES areas, e.g., a low-fiber and high-calorie diet, which is associated with a high incidence of CRC. Future studies should assess the relationship between SES and the incidence of CRC.

Population-based CRC screening, including fecal occult blood test and colonoscopy, is effective for early diagnosis of CRC and therefore may reduce the CRC incidence and CRC-related mortality [23]. Based on the data from five randomized controlled trials involving 404,396 participants, 2–9 rounds of biennial screening using the guaiac-based fecal occult blood test (Hemoccult II) compared with no screening resulted in a 9%–22% reduction in CRC-specific mortality over 11–30 years of follow-up [24]. From 2004 to 2013 in the US, the CRC incidence decreased by an average of 1.4% per year among individuals aged 50–64 years and by 4.0% per year among those aged ≥65 years [25]. This is likely because the rate of participation in colonoscopy screening increased from 14% in 2000 to 41% in 2013 among individuals aged 50–54 years, from 16% to 52% in those aged 55–59 years, and from 25% to 63% in those aged ≥65 years [25]. In Guangzhou, the CRC screening program was implemented in Yuexiu District in 2010 and was extended to all Districts in 2015. The screening program was promoted by the media since 2013.

The results of this study have important public health implications. Identification of the spatial and temporal distribution of CRC incidence provides not only information regarding cancer etiology for primary prevention but also reference for appropriate allocation of health resources. Further genetic, environmental, and socioeconomic investigations will enhance our knowledge of the causes and epidemiology of CRC. Primary prevention and screening programs should focus on central urban high-risk areas to improve CRC control.

In this study, we applied spatio-temporal analysis to identify variations in the distribution of CRC. A few limitations should be noted. First, our spatial and temporal analyses were based on CRC incidence data, which could be affected by differences in diagnostic techniques among hospitals, access to health care, cancer registration quality, and so on. Second, because of the lack of information on risk factors among streets, no covariates were considered in the analysis. Third, the street-level population data were derived from the 2010 census and based on known permanent addresses, which could have been inaccurate. Therefore, the results of this study should be interpreted with caution.

## Conclusions

In summary, this study identified clusters of areas with high CRC incidence in Guangzhou, specifically in the central urban areas (including the border regions of Baiyun, Haizhu, Liwan, and Yuexiu Districts). CRC screening programs should focus on those areas, and factors related to the spatio-temporal pattern of CRC incidence in Guangzhou should be investigated.
